# A model for estimating costs and benefits of new vaccine technologies from the perspective of both buyers and sellers

**DOI:** 10.1371/journal.pone.0283977

**Published:** 2023-04-05

**Authors:** Michael Krautmann, Ben Davis, Pascale R. Leroueil

**Affiliations:** William Davidson Institute, University of Michigan, Ann Arbor, MI, United States of America; University for Development Studies, GHANA

## Abstract

Although vaccination is widely considered one of the most cost-effective health interventions available, global coverage rates for many vaccines remain lower than necessary for disease elimination and eradication. New vaccine technologies can play an important role in addressing barriers to vaccination and increasing coverage rates. To identify and prioritize vaccine technology investments, decision makers must be able to compare the overall costs and benefits of each investment option. While these data points may exist, they are often confined to silos. Decision makers would benefit from a model that synthesizes this broad range of data and provides clear and actionable information. To facilitate vaccine investment, purchasing and deployment decisions, we developed a systematic and transparent cost-benefit model that estimates the value and risk of a given investment scenario from the perspective of both “buyers” (e.g., global donors, country governments) and “sellers” (e.g., developers, manufacturers) of vaccines. This model, which can be used to evaluate scenarios related to a single vaccine presentation or a portfolio of vaccine presentations, leverages our published approach for estimating the impact of improved vaccine technologies on vaccination coverage rates. This article presents a description of the model and provides an illustrative example application to a portfolio of measles-rubella vaccine technologies currently under development. Although the model is generally applicable to organizations involved in vaccine investment, manufacturing or purchasing, we believe it may be particularly useful to those engaged in vaccine markets that rely strongly on funding from institutional donors.

## Introduction

Although vaccination is widely considered one of the most cost-effective health interventions available [1], global coverage rates for many vaccines remain lower than necessary for disease elimination and eradication [2]. In an effort to address this gap, organizations such as the Bill & Melinda Gates Foundation and Gavi, the Vaccine Alliance, invest in developing new vaccine technologies that can improve coverage by ensuring more effective, efficient, and equitable vaccine delivery. Given the finite resources available, these investments must be prioritized in some manner.

Any prioritization process can be quantitative [3], qualitative [4] or a combination of the two [3]. Fundamentally, however, the process is a type of cost-benefit analysis (CBA) that consists of weighing the costs and benefits of a potential investment relative to the costs and benefits of other potential investments. Within the global health space, the Vaccine Innovation Prioritization Strategy (VIPS) is the most prominent prioritization process for vaccine-related investments [5]. The prioritization process was informed by consultations with key stakeholders [6], technology developers, industry representatives, regulators and international agencies [7]. These consultations provided a qualitative picture of the benefits and costs of each potential vaccine investment. In addition, they surfaced a substantial amount of quantitative data related to benefits and costs [8]. However, to our knowledge, there was no quantitative CBA completed allowing decision makers to compare the relative value of each potential vaccine investment or optimize an overall portfolio of investments.

There are several potential reasons that groups operating in the global vaccine market, including VIPS, may not complete quantitative CBAs to inform their investment decisions. *First*, we are not aware of a publicly available CBA model that is tailored to vaccine-related investments. While there are disease specific CBA models tailored to disease interventions [9], they are not obviously applicable to vaccine-related investments. *Second*, we are not aware of a publicly available CBA model that can be used for existing, *and* still-in-development health-related technologies. Indeed, the models we are aware of are entirely focused on existing health-related technologies. *Third*, we are not aware of a publicly available CBA model for health-related technologies that captures the perspective of both buyers and sellers, which we believe is particularly important when considering investments in earlier-stage technologies since users may build a plan around an unrealistic expectation (e.g., unit cost, potential demand). *Fourth*, we are not aware of a publicly available CBA model that includes the ability to incorporate uncertainty into key inputs. This would be useful for providing the user some sense of ‘risk’ related to the investment, but particularly important for still-in-development health-related products. *Fifth*, we are not aware of a publicly available CBA model that allows users to determine the most cost-beneficial portfolio. Taken together, we believe a transparent CBA model that captures the expected costs, benefits and uncertainties of still-in-development vaccine technologies from the perspectives of both vaccine buyers and sellers may be useful to those operating in the global vaccine market.

In this article, we first present the two conceptual components of our model: a CBA framework and a portfolio selection analysis framework. Next, we describe how we defined and modeled the costs, benefits and risks associated with “buyers” (e.g., global donors, country governments) and “sellers” (e.g., developers, manufacturers) of vaccines. We show the type of output generated from the CBA of a single vaccine presentation and then describe how the model can be used to identify the most cost-effective portfolio of vaccine presentations from the perspective of a buyer. After describing our implementation in Microsoft Excel, we summarize an example portfolio analysis that uses a set of nascent measles-rubella vaccine technologies. Finally, we discuss the benefits and limitations of our model.

Our aim was to develop a systematic and transparent cost-benefit model to estimate the value and risk of a given investment scenario from the perspective of both buyers and sellers of vaccines. We achieved this aim in terms of producing an Excel-based model to provide a quantitative financial assessment of a potential investment scenario, both for a single vaccine investment and a portfolio of vaccine investments. We believe this model, which leverages our method for estimating the impact of new vaccine technologies on vaccination coverage rates [10], would be useful to organizations involved in vaccine investment, manufacturing or purchasing. It may be particularly useful to parties working in vaccine markets that are historically less profitable (e.g., global health donors), including those engaged in VIPS.

## Description of model

Our model consists of two main components designed to address the key use cases of potential investors in vaccine technologies:

1. A **CBA framework** that, for a single vaccine technology, aims to help users better understand the conditions (e.g., vaccine price, demand, development cost) in which that technology would be attractive to potential vaccine buyers and sellers.
2. A **portfolio selection analysis framework** that combines outputs from the CBA and our vaccination coverage method [10] to identify which combinations of vaccine technologies maximize coverage rate at the lowest total cost.

**Fig 1:**
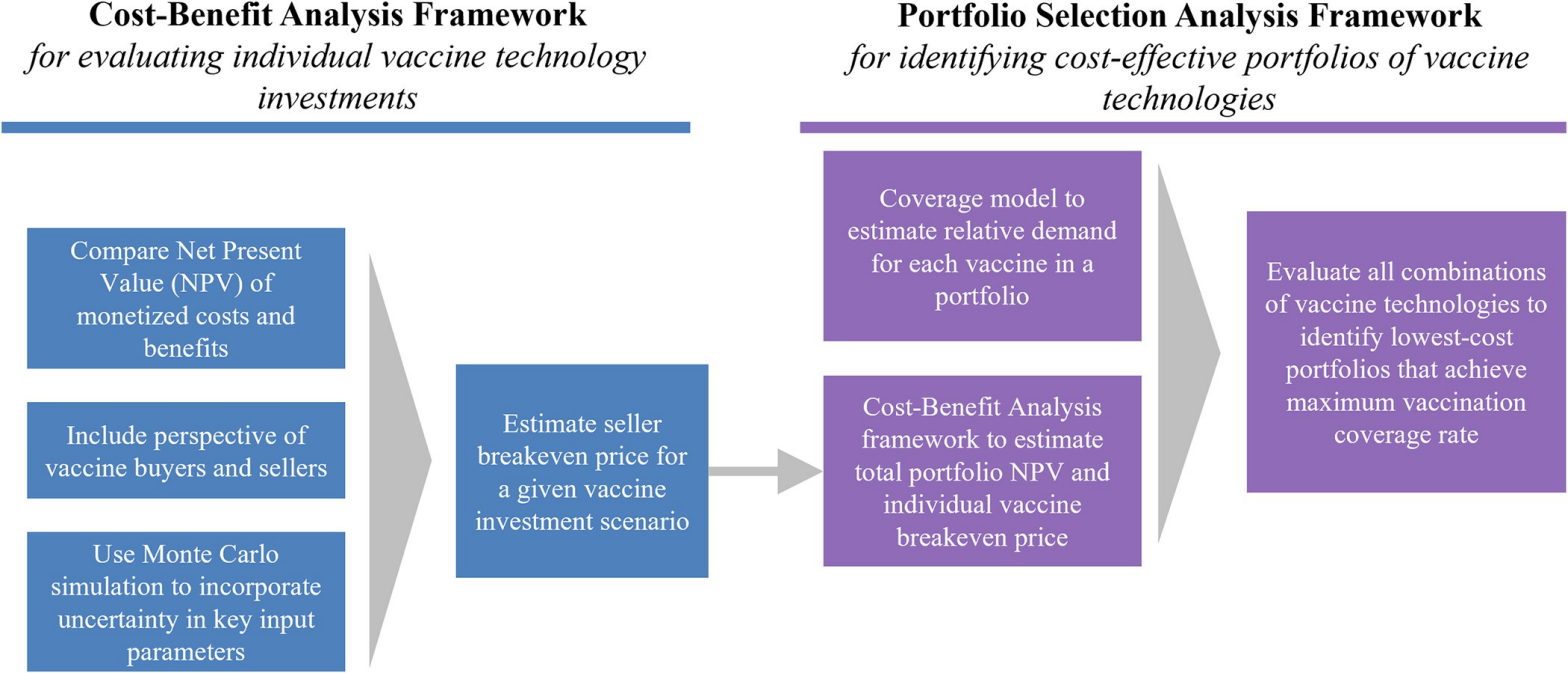
Conceptual overview of the model for evaluating the costs and benefits of potential vaccine investments, both individually and as a portfolio of multiple potential investments.

The relationship between these two components is shown in Fig 1.

In this section we describe the overall structure of the CBA framework, including specific components of cost and benefit that feed into a Net Present Value (NPV) calculation for buyers and/or sellers. Next, we discuss additional layers of complexity that must be incorporated into the basic cost-benefit analysis structure, such as accounting for uncertainty in key parameters and solving for unknown input parameters like expected unit price. We also describe how this basic cost-benefit analysis structure can be incorporated into a broader portfolio selection analysis. Finally, we briefly summarize how we implemented this model as an Excel-based tool and present an example analysis.

### Cost-benefit analysis framework

The first main component of the model is a cost-benefit analysis focused on a single vaccine development and delivery scenario. We focus on a single vaccine buyer and a single vaccine seller, each of whom face independent but interrelated cost-benefit decisions about whether to move forward with a particular vaccine investment or purchase.

***Vaccine buyers*** typically consist of donor agencies and/or country governments, which are responsible for funding vaccine development efforts as well as purchasing and delivering finished vaccine doses. Because these parties have a common objective of maximizing societal benefit through increased vaccination coverage rates [11], we can generally treat them as a single entity.

***Vaccine sellers*** typically consist of manufacturers who develop, commercialize, and produce a vaccine presentation to sell to vaccine buyers. Sellers typically do NOT share a common objective. Each seller seeks to maximize its own benefit rather than societal benefit. In the case where multiple manufacturers operate in parallel, we must focus on one seller at a time.

At least one willing buyer and seller are needed for a particular vaccine technology to be successfully developed and deployed. By capturing both buyer and seller cost-benefit decisions in the same overarching model, the CBA framework helps clarify the conditions (e.g., development cost, unit price, demand volume) in which both parties would likely be willing to invest in a given vaccine technology.

For both buyers and sellers, the cost-benefit analysis calculation involves estimating and monetizing total costs and benefits of a vaccine technology over a specified future period and consolidating those elements into an NPV using the following equation [12]:

$$NPV=\sum_{t=1}^{T}\frac{B_{t}-C_{t}}{{\left(1+i\right)}^{t-1}}$$ (1)

where:

B_t_ = sum of all buyer or seller benefits accrued in year t

C_t_ = sum of all buyer or seller costs accrued in year t,

i = buyer or seller cost of capital, i.e., the percentage rate at which future costs or benefits are discounted to bring them into present value dollars

T = the number of years included in the buyer’s or seller’s analysis

NPVs are calculated independently for buyers and sellers. An NPV greater than zero implies that the present value of the benefits exceeds the present value of the costs, making the vaccine technology a good investment for the buyer or seller conducting the analysis. An NPV less than zero, on the other hand, implies that the vaccine technology is *not* a good investment for the buyer or seller. A change to one or more of the cost or benefit inputs (e.g., a change in unit price, expected demand, or other estimates and assumptions) would be needed to change the NPV.

#### Overview of benefits and costs for vaccine buyers and sellers

**Fig 2:**
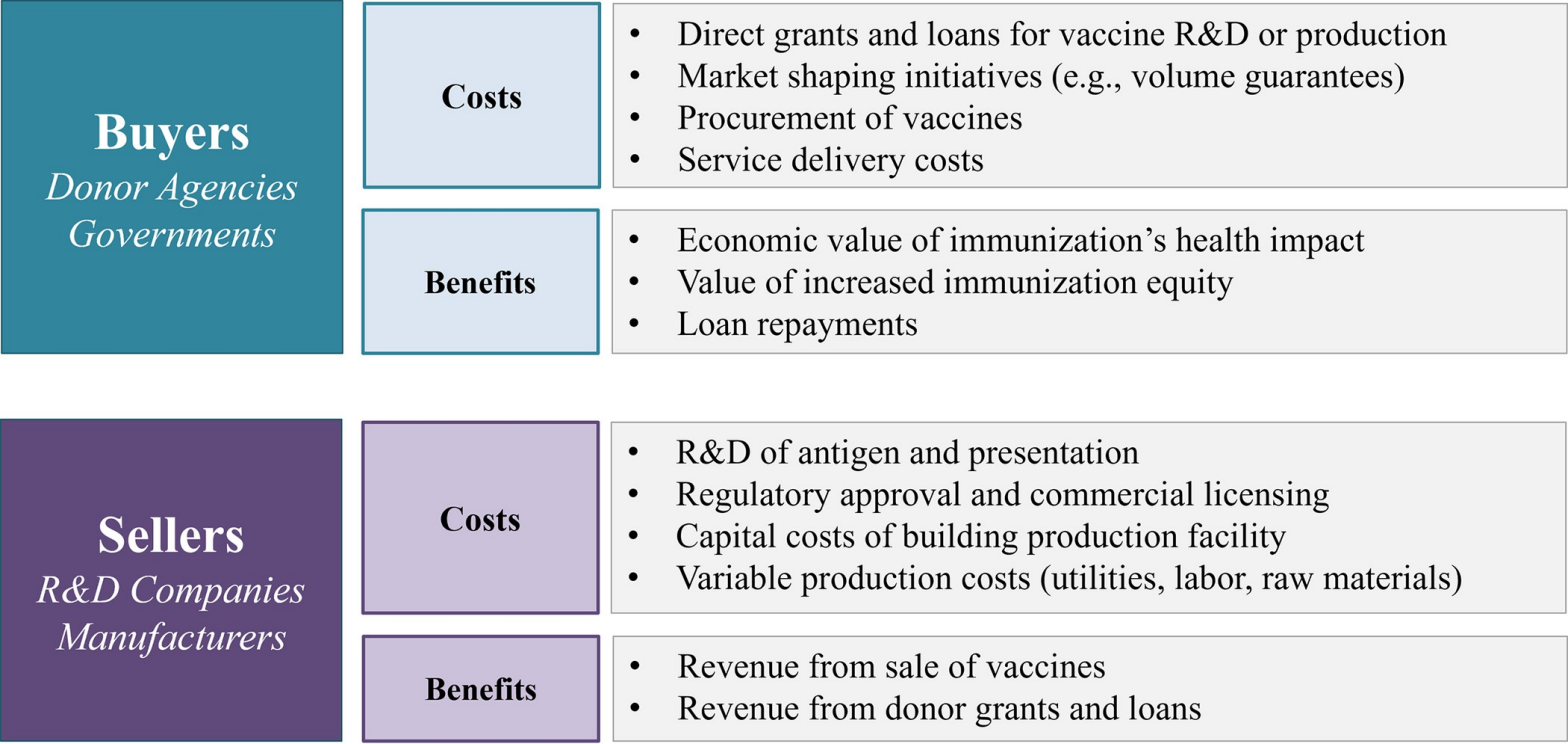
Summary of key costs and benefits for vaccine buyers and sellers.

Vaccine buyers and sellers share an independent but interrelated set of costs and benefits, summarized in Fig 2.

For vaccine buyers, key costs include the purchase of vaccine doses, delivery of doses to the target population, and any investments or loans made to support research and development, market shaping, demand generation, or other related interventions [13, 14]. Buyer benefit is driven primarily by the number of people who are immunized against target diseases, the health impact gained from that immunization, and ultimately the economic value associated with that health impact. Therefore, buyer benefits are affected by several vaccine characteristics, like efficacy rate and the probability of technical and regulatory success, as well as population characteristics like disease incidence rate and vaccination coverage rate.

For vaccine sellers, key costs are those associated with developing and commercializing a vaccine candidate as well as the fixed and variable costs of producing vaccine doses sold (e.g., capital expenditures to build a production facility, materials and labor to produce each vaccine dose) [15]. Seller benefit derives primarily from the sale of vaccine doses to buyers. Thus, key parameters affecting seller benefit include the vaccine unit price, the size of the target population (i.e., potential demand for vaccine doses), and the seller’s expected market share. Sellers also benefit from any grants received during the development or production process. A more detailed list of input parameters used to calculate costs and benefits can be found in S2 File. Importantly, many of these parameters are included in both buyer and seller cost-benefit calculations. For example, vaccine unit price is both a key driver of benefit for sellers and a key driver of cost for buyers. Target population size and growth rate is the main driver of demand, thereby affecting costs and benefits for both sellers and buyers.

Additionally, costs and benefits must be considered over a fixed future time horizon, e.g., 10 years. This time horizon does not need to be the same for buyer and seller. For example, vaccine manufacturers may require a positive NPV within a few years of initial production, while donors and other buyers may consider a time horizon of 10 years or more when making investment decisions. Future costs and benefits are also subject to the buyer’s and seller’s assumed cost of capital, the rate used to discount costs and benefits that are realized in the future, and the expected annual inflation rate in target population geographies. Again, these values do not need to be the same for buyer and seller.

#### Modeling key cost-benefit analysis parameters

The previous section describes the basic structure of a buyer and seller CBA for vaccine technology investments. However, this description excludes several factors, such as uncertainty, pricing, and risk tolerance that are critical for replicating a real-world decision-making context. In this section we describe how our approach incorporates these elements to create a more realistic decision-making model.

*Using Monte Carlo simulation to incorporate uncertainty*. Both vaccine buyers and sellers face significant uncertainty in many of the key costs and benefits that factor into vaccine investment decisions. To account for this uncertainty, the model uses Monte Carlo simulation to represent several input parameters as probabilistic variables rather than deterministic values:

- Probability of technical and regulatory success: Not all vaccine candidates successfully achieve regulatory approval and reach production. Our model includes six research and development (R&D) stages, each with a binary “succeed or fail” outcome and an independent probability of success. A vaccine candidate must succeed in all six stages to progress to manufacture and commercialization. Failure has several implications in the model. No doses are produced and sold by manufacturers, and no doses are delivered by buyers, resulting in no net benefit for either party. Manufacturers still incur R&D costs that vary based on the stage of failure, but not fixed or variable production costs. Buyers incur the cost of any grants made before the year in which production was expected to begin.
- R&D and capital investment costs: In addition to the uncertainty of technical and regulatory success, the true cost of R&D and facility capital investment also varies depending on factors like the size and duration of clinical trials or the complexity of manufacturing processes. Thus, these parameters are captured in the model as normally distributed random variables, with a mean (expected value), and a standard deviation expressed as a percentage of the mean. For example, a parameter might have an expected cost of 100 million dollars and a standard deviation of 25%, or 25 million dollars.
- Annual demand for vaccine doses: Both buyers and sellers face uncertainty in future demand for vaccine doses, driven by factors like country demand generation and competitive bidding processes. As with the R&D costs above, the model captures annual demand as a normally distributed random variable with two input parameters: expected demand and standard deviation expressed as a percentage of expected demand. Expected demand is modeled as a function of target population size, vaccination coverage rate, number of doses needed for immunization, and open- and closed-vial wastage assumptions.
- Service delivery costs: For buyers, service delivery costs are a large part of vaccines’ total costs, but existing research to quantify service delivery costs is relatively nascent. They are captured in the model as a normally distributed random variable with a mean (expected value) and a standard deviation expressed as a percentage of the mean.

These uncertain parameters are incorporated into the model by (1) randomly sampling from each parameter’s distribution to determine specific input values, (2) using those specific inputs to perform the remaining buyer and seller NPV calculations described in the previous section, and (3) replicating the first two steps numerous times to estimate the probability distribution of buyer and seller NPVs [16].

*Estimating seller breakeven price*. The selling price of a vaccine unit is a critical input for calculating buyer costs and seller benefits. However, in the context of vaccine investments, this value is often unknown to potential buyers [17]. Our model treats Unit Price as an unknown variable within the basic CBA framework and uses a heuristic to estimate the seller’s “breakeven price”, i.e., the lowest vaccine unit price such that the seller NPV is greater than or equal to zero. This breakeven price is assumed to be the lower bound of the vaccine’s actual selling price.

The model uses a two-stage heuristic to solve for breakeven price to the nearest $0.01. We originally structured the problem as an optimization that used Excel Solver, but Solver proved too computationally intensive. The model’s heuristic works as follows:

1. Starting from $0, Unit Price increases in $1 increments, and simulation is refreshed each time to check if seller NPV > = 0. Once this is true (say at $N), this first step in the algorithm stops. We now know that the breakeven price is between N-1 and N.
2. Next, the model uses a binary search algorithm [18] to efficiently search the space between N-1 and N for the point at which Seller NPV = 0. This algorithm iterates until the search interval is less than $0.01; the upper bound of this remaining interval is assumed to be the breakeven price.

Our model assumes that pricing is based on the actual cost of the vaccine and does not factor in competitive pricing tactics such as loss leaders, cross-subsidization, or pricing to what the market will bear. It also does not explicitly include a profit margin, although the broader model includes several elements that typically affect margins such as cost of capital and perceived risk.

*Capturing differences in buyer and seller risk tolerance*. As described above, each replication of the Monte Carlo simulation results in a unique NPV outcome due to the inclusion of randomly sampled probabilistic variables. A single set of user inputs (i.e., a set of defined distributions for all probabilistic variables) may yield a wide range of potential outcomes, including both negative and positive NPVs. In other words, any potential investment scenario carries some risk of resulting in a negative NPV, and different buyers and sellers may have different levels of tolerance for this risk [19, 20].

We define risk tolerance as a required percentage of positive simulated outcomes (i.e., outcomes in which NPV is greater than $0). For example, a conservative buyer or seller might want to see a positive NPV in at least 90% of simulated outcomes before they are willing to make a deal. A more risk-tolerant buyer or seller may be happy if only 20% of simulated outcomes are positive, so long as the average NPV is still positive.

The model implements this concept by including risk tolerance thresholds, ranging from 0% to 100% (non-inclusive), as independent input parameters for both buyers and sellers. This risk tolerance threshold is used to determine a relevant percentile value from the range of simulated NPV outcomes. For example, if a seller has a risk tolerance threshold of R%, then they will accept a negative seller NPV outcome in at most (1-R)% of the simulation runs. Therefore the (1-R)^th^ percentile value among the simulation runs must be greater than or equal to zero for the seller to agree to the terms of the simulation scenario. Note that based on this definition, risk tolerance threshold and percentile value are complementary–they sum to 100%.

*Estimating and modeling key health economics inputs*. The cost-benefit analysis framework includes several input parameters that draw from the field of health economics and are needed to define the health impact and monetary benefit of vaccination to buyers. In this section we describe how these parameters are defined and integrated into the model.

We measure health impact in disability-adjusted life-years (DALYs) averted through immunization. In the model, this parameter contains two parts that are multiplied according to the following equation:

$$DALYs\;Averted\;per\;Person=\sum_{j=1}^{J}\left[C_{j}\times D_{j}\right]$$ (2)

where:

C_j_ = the number of cases of disease *j* averted per fully vaccinated person

D_j_ = the number of DALYs averted per disease case averted for disease *j*

J = the set of diseases against which the vaccine in question offers protection

The parameter C_j_, which represents the number of cases averted per person in the above equation, is defined by the following equation:

$$C_{j}=E_{j}\times I_{j}\times P_{j}$$ (3)

where:

E_j_ = vaccine efficacy against disease *j*, expressed as a percentage between 0% and 100%

I_j_ = annual incidence rate of disease *j* in the target population, expressed as a percentage of the target population expected to contract the disease within a given year

P_j_ = duration of protection of the vaccine in question against disease *j*, expressed in number of years. For vaccines with lifetime protection, duration of protection is defined as the average age of vaccination subtracted from the population’s weighted average life expectancy.

The parameter D_j_, the number of DALYs averted per disease case averted, can be calculated in one of two ways. The preferable way is to use well-established existing data sources that employ rigorous epidemiological modeling. For example, this value can be estimated using Global Burden of Disease data [21] by dividing Total DALY Burden by Total Incident Cases for a given disease, population, and year.

However, for diseases not covered by existing datasets, our model provides a simple method for estimating the parameter D_j_. While it is aligned with common methods for estimating the health impact of vaccination [22, 23], our method does not attempt to capture the full complexity of an epidemiological model. It estimates the two DALY components, Years of Life Lost per case (YLL) and Years Lived with Disability per case (YLD), using the following formulas:

$$D_{j}={YLL}_{j}+{YLD}_{j}$$ (4)

$${YLL}_{j}={CFR}_{j}\times [L-A_{j}]$$ (5)

$${YLD}_{j}=\sum_{k=1}^{3}\left[W_{k}\times F_{jk}\times S_{jk}\right]$$ (6)

where:

CFR_j_ = case fatality rate of disease *j*, expressed as a percentage of cases resulting in death

L = life expectancy of the average person in the target population, expressed in years

A_j_ = average victim age at the time of death for disease *j*

W_k_ = disability weight for condition *k*, expressed as a number between 0 and 1, representing the seriousness of a disability caused by one of the diseases in question. It is equivalent to the fraction of each year lost due to living that year with a particular disability.

F_jk_ = frequency with which disability condition *k* occurs in cases of disease *j*

S_jk_ = average symptom duration (in years) for disability *k* when caused by disease *j*

K = the set of disability conditions caused by disease *j*. The model provides space for up to three conditions per disease, although the model does not require any conditions to be entered if the user wishes to leave out the YLD term. For most vaccine-preventable illnesses the value for YLL is substantially higher than the value for YLD.

Determining the economic value of this health impact is critical to quantifying the benefit that buyers receive from their purchases and investments, and is a relatively nascent area of research for vaccination, especially in low-and-middle- income countries [24, 25]. The model does not propose a particular approach for estimating the economic value of vaccination-related health outcomes. The model uses only a single parameter, Baseline Benefit per DALY Averted. It includes optional reference values as a potential guide and resource to users. These values are based on research by Robinson et al., [26] which proposes a method for extrapolating Value of a Statistical Life Year (VSLY) estimates from high-income countries to a low-and-middle-income (LMIC) country context. Using these estimates in our model requires an assumption that a DALY and a Statistical Life Year are functionally equivalent.

*Equity considerations for costs and benefits*. Up to this point, our description of the model implies that every individual within the target population is identical in terms of the health impact and economic value gained from vaccination as well as the cost to vaccinate that individual. However, we know that this is not true in practice. Certain population segments are objectively more expensive to reach, such as remote rural populations that require costlier transportation, or underserved sub-groups with less access to the health system, who require extensive outreach by health workers. Many of these same population segments are also a high priority for vaccine buyers; donors and governments are often willing to fund more expensive technologies or interventions to reach these sub-populations [27], implying a greater buyer-side benefit.

Though research to quantify differences in vaccine delivery cost or economic benefit across specific sub-groups is limited, the model provides a basic framework for incorporating these differences. Individual sub-groups can be defined, each representing a percentage of the overall target population. Each sub-group can be assigned a multiplier for both service delivery cost per dose (buyer cost) and value per DALY averted (buyer benefit). The multiplier is then applied to the baseline value for the overall population. For example, if baseline service delivery cost is $2.00 per dose and a particular sub-group has a cost multiplier of 1.3, the service delivery cost per dose for that sub-group is assumed to be 1.3 x $2.00, or $2.60. In this way, the model is intended to provide flexibility to incorporate user estimates or future research as it becomes available.

#### Example output from cost-benefit analysis

The output of this CBA framework is a distribution of possible NPV values, for both buyers and sellers, for each year of the analysis. Table 1 shows a simple conceptual example of how the CBA outputs for the two parties can be visualized.

**Table 1:** Conceptual example of the output from the above CBA framework. Note that the framework will result in two sets of outputs–one for sellers, one for buyers–as depicted above.

	**Seller Net Present Value (NPV) by Percentile**			**Probability of NPV > 0**
	*Illustrative amounts in million USD*			
	10^th^	50^th^	90^th^	
***After 1 year***	-$5	-$1	$20	40%
***After 2 years***	-$6	$3	$40	55%
***After 3 years***	-$7	$5	$60	60%
***After 4 years***	-$8	$10	$80	65%
***After 5 years***	-$10	$15	$100	70%
	**Buyer Net Present Value (NPV) by Percentile**			**Probability of NPV > 0**
	*Illustrative amounts in million USD*			
	10^th^	50^th^	90^th^	
***After 1 year***	$0	$3	$40	60%
***After 2 years***	$0	$6	$80	75%
***After 3 years***	$0	$10	$120	80%
***After 4 years***	$0	$20	$160	80%
***After 5 years***	$0	$30	$200	80%

With this output the user can determine several values relevant for decision-making, such as the median and mean NPV after *n* years, the likelihood of a positive NPV after *n* years, and the range of NPV outcomes broken down by percentile. The percentile breakdown allows users with a wide range of risk tolerances and investment timeframes to evaluate the same investment scenario. For example, if a particular seller required a 90% or greater likelihood of a positive return on investment within 5 years, they would look for the 10th percentile NPV (i.e., 100% - 90%) to be positive at the 5-year mark. In the visualization above, this seller NPV value is negative, indicating that the seller would not likely choose to invest, and some input parameter (e.g., price, demand volume) would need to be adjusted until the 10^th^ percentile NPV was positive.

### Portfolio selection analysis framework

Thus far our description of the CBA framework has focused on a basic use case: helping buyers or sellers evaluate a *single* vaccine candidate by estimating an important parameter like unit price. However, in many cases, buyers are evaluating and prioritizing *multiple* new vaccine candidates for a given disease area. These buyers seek to identify portfolios of vaccine presentations (i.e., quantities of each presentation delivered to the target population) that maximize overall buyer value. Our objective with portfolio selection analysis is to find the lowest-cost global portfolio option that maximizes global coverage rate.

#### Estimating demand for each vaccine in a portfolio at the country level

**Fig 3:**
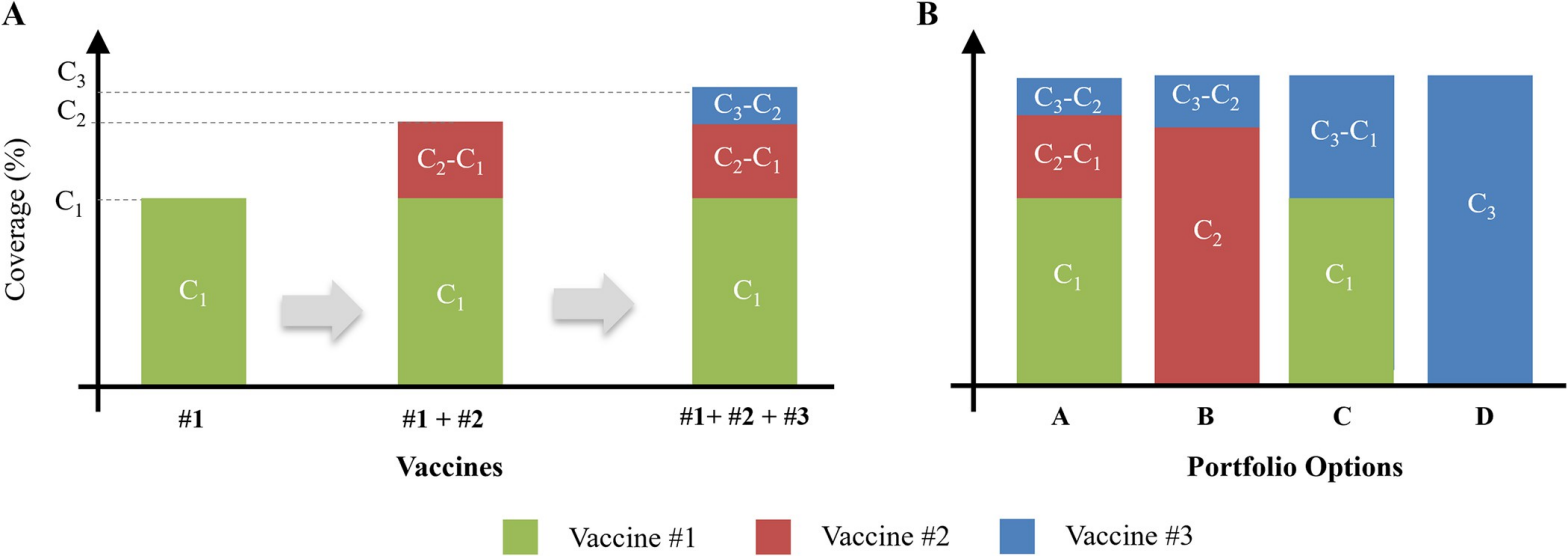
Conceptual diagram showing how the authors’ previously published coverage model can be used to estimate the relative proportion of each presentation in a portfolio. Panel A shows how adding vaccines sequentially results in a marginal increase in coverage. Panel B shows how changing the order in which vaccines are added results in different portfolio compositions.

Addressing this portfolio selection use case requires a way to estimate the number of doses needed for each vaccine presentation within a given portfolio. To accomplish this, we leveraged our published method [10] for estimating the impact of vaccine technologies on vaccination coverage rates. This method estimates how well a new vaccine technology addresses known barriers to vaccination such as limited access to cold chain equipment or trained personnel. It calculates the marginal change to current coverage rates should that technology be introduced in place of *or in addition to* vaccine presentations already available. This output from our coverage model can be used iteratively to estimate the relative proportion of each vaccine in a portfolio, as shown in Fig 3.

The figure shows a set of three vaccine presentations that are considered for a hypothetical portfolio. We estimate the relative proportion of each vaccine by “adding” them one-by-one to a portfolio, using the coverage model estimates to calculate *the marginal* increase in expected coverage rate over other vaccines already in the portfolio. To achieve a nonzero marginal increase, the newly added vaccine must improve upon the existing portfolio of vaccines in some dimension (such as greater thermostability or fewer doses per container). We assume that each vaccine’s marginal increase represents the portion of the target population that would be vaccinated using that vaccine. It is a proxy for the relative demand for that vaccine in the portfolio.

However, this marginal increase changes depending on the order in which the vaccines are added. A single set of vaccine presentations can yield multiple possible portfolios. This effect is illustrated on the right side of Fig 3, where different permutations of the same three vaccines yield portfolios with widely varying demand compositions. The output of this method–the proportion of a target population vaccinated using Vaccine *X*–can be used as an input to the CBA framework and combined with other inputs (such as closed and open-vial wastage) to estimate true demand for doses of Vaccine *X* for a given portfolio option.

#### Aggregating country demand into global-level demand

**Fig 4:**
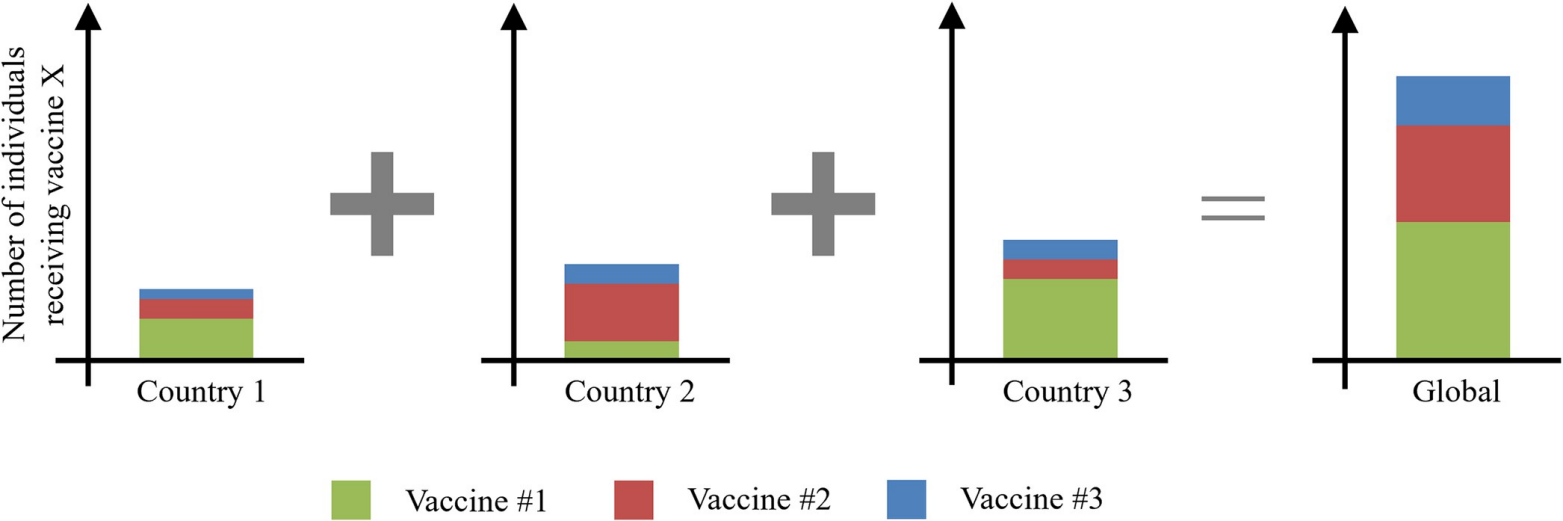
Conceptual diagram of the aggregation of several country-level portfolios into a single global-level portfolio, for a given vaccine ordering scenario (in this case Vaccine #1 → Vaccine #2 → Vaccine #3). Individual country demand for each vaccine presentation is calculated using the country-specific portfolio distribution and total eligible population. Country-level demands are then summed to yield the global demand for each vaccine presentation.

In many cases, the above demand estimation approach will be applied at a country level since most of the required data sources report on an individual country basis. However, many vaccine buyers—especially donor agencies and investors—are concerned with *global level* demand for a particular vaccine technology. In most cases global demand is the primary driver of buyer benefit and expected unit price. Therefore, our model must aggregate a series of country-specific vaccine portfolios into one overarching global portfolio, as depicted in Fig 4.

For each country, the most cost-efficient way to build a portfolio using the above approach is to add the vaccine presentations in increasing price order. This ensures that as much demand as possible is allocated to the lowest cost vaccines, and demand for more expensive vaccines is limited to marginal coverage increases over lower cost options. We assume that vaccine price is generally a function of *global* demand, so the optimal price order will be the same across all countries in the analysis.

However, the optimal price order cannot be determined *a priori*, since vaccine price, global demand, and individual country demand have a circular relationship. Therefore, we use a full factorial approach to enumerate and test all possible price ordering permutations (“portfolio options”) to determine which one is optimal at a global level. In other words, we seek to answer the question, “Of the portfolio options that maximize global coverage rate, which one(s) have the lowest total buyer cost?” Our approach is as follows:

1. Determine all possible price-order permutations for a given set of vaccine presentations
2. For each permutation:
  - Loop through each country in the analysis, applying our coverage rate estimation methodology [10] to each successive vaccine presentation to estimate the marginal coverage rate increase attributable to that vaccine.
  - For each vaccine presentation, aggregate demand across all countries by multiplying each country’s target population by the vaccine’s marginal coverage rate for that country.
  - Apply the CBA framework to each presentation using aggregate demand as an input variable to estimate expected unit price and total buyer benefit/cost
  - Sum total buyer benefit and cost across all presentations
3. Once all permutations are complete, identify the set of permutations that yield the highest buyer benefit. This should correlate with maximum global coverage rate. Of that set, identify the permutation with the lowest total buyer cost.

This approach identifies the optimal portfolio option, and in doing so estimates total cost, total benefit, and global and region-specific demand for each vaccine presentation in the portfolio.

### Excel implementation

We implemented the above method as a model in Microsoft Excel (S1 File). The model is comprised of five main components:

- **Setup & Results Worksheets** allow the user to input parameters for analysis and display the results of analysis.
- **Key Data Sources Worksheets** capture most of the input data used for analysis.
- **Detailed Calculations Worksheets** contain the formulas used to transform input data into analysis results. This component includes worksheets from the Excel implementation of our previously published method for estimating coverage impact [10].
- **Other Reference Worksheets** contain additional inputs and tables referenced by calculation worksheets.
- **Visual Basic for Applications** code (1) transfers data between the above worksheets, (2) generates the full set of portfolio options, and (3) estimates seller breakeven price using the algorithm described previously.

Additional detail on each worksheet is provided in the User Guide of the supplement file.

### Validation

In our previous publication we discussed challenges in gathering sufficient data to fully validate the coverage rate estimation methodology [10]. Because that methodology is critical to estimating the relative demand for vaccines in a portfolio, those same validation challenges also apply to the methodology described here.

Furthermore, the cost-benefit and portfolio selection analyses have additional data needs that complicate the validation process. On the seller side of the CBA framework, key data sets include a full accounting of fixed and variable development and production costs, actual cost of capital figures, and actual historical prices, most of which are proprietary and inaccessible to the public. On the buyer side, key data sets include actual service delivery costs, research and development investment totals, and historical examples of NPV or cost-benefit analyses being applied to vaccine technologies. Most of these data sets either do not exist or exist only for small, isolated examples (such as a single country or vaccine presentation). Because of this, we were unable to locate a single target population and presentation for which all the required data elements were present.

Despite a lack of data to fully validate the model, we sought expert feedback from both buyer and seller perspectives on the overall structure and conceptual approach. On the buyer side this included representatives of Gavi’s Market Shaping team and the Bill & Melinda Gates Foundation’s Vaccines Development team. On the seller side, we engaged several individuals and grantees of the Gates Foundation’s Chemistry, Manufacturing, and Control (CMC) team with experience at vaccine development and manufacturing organizations. We used feedback from these experts to refine both the structure of, and inputs to, the cost-benefit and portfolio selection analyses.

## Example application of model

### Analysis setup

As a proof of concept, we applied the model to a set of five potential measles-rubella (MR) vaccine presentations. Three of these presentations are new vaccine technologies currently under development with funding from the Bill & Melinda Gates Foundation. They include:

1. A **microarray patch (MAP)**, which offers improved thermostability, administration by minimally trained personnel, and the possibility of improved patient acceptability due to the lack of a needle.
2. A **dual-chamber device**, which integrates vaccine and diluent in separate compartments of a syringe to simplify reconstitution. It offers the possibility of increased thermostability and lower training requirements for administration.
3. **Aerosol delivery**, which also has the potential to improve thermostability and reduce training requirements for administration.

Because these technologies are still under development, we included two different presentations for each: a version with “minimum acceptable” characteristics and a version with “optimal” characteristics. In the case of MR-MAP, these presentations are defined based on published Target Product Profiles [28]. In the case of dual chamber and aerosol devices, the presentation characteristics are based on expert projections from the Gates Foundation CMC team.

Additionally, we included two existing presentations–a 10-dose vial and a 5-dose vial produced by Serum Institute of India and prequalified by the World Health Organization [29] –which are administered subcutaneously and require cold storage in 2–8 degrees Celsius. These two presentations, together with the three new technologies above, form a potential portfolio of five MR vaccine presentations with which to conduct a portfolio selection analysis.

The scope of this analysis is all 82 countries classified as low- or lower-middle-income by the World Bank for its 2023 fiscal year [30]. Collectively, these countries have an annual birth cohort of 94 million children per year and a first-dose coverage rate of 81.2% for measles-containing vaccines. We assume that the current baseline presentation for all countries is the 10-dose vial, and that the overall timeline for analysis is 10 years.

We collected inputs from numerous sources for this analysis. A brief description of these sources is available in S2 File. Broadly speaking the required inputs fall into three categories:

1. **Target population characteristics**, including target population size and demographics; disease incidence and fatality rate; vaccination coverage rate; service delivery costs; and equity characteristics of key sub-populations.
2. **Vaccine presentation characteristics**, including Vaccine Scores for each of the five technology addressable barriers from our previously published coverage estimation methodology; estimated probability of technical and regulatory success; estimated fixed and variable costs of production; number of doses required; closed and open-vial wastage rates; and relative impact on service delivery cost as a percentage of baseline target population costs.
3. **Health economics inputs**, which primarily include the health impact and economic value of a disease case averted through immunization, as well as the economic value multipliers for key sub-populations.

As mentioned previously, many of these inputs lack definitive or publicly accessible values, particularly for the three new vaccine technologies. Therefore, our dataset relies on expert judgment and secondary analysis of existing data.

### Results and key findings

Because the new technologies are still in the early stages of development, this analysis was intended as a broad overview of the MR vaccine market rather than a detailed comparison of specific technology options. In this section, we describe several insights about the composition of a future MR portfolio that can be drawn from a portfolio selection analysis.

**Fig 5:**
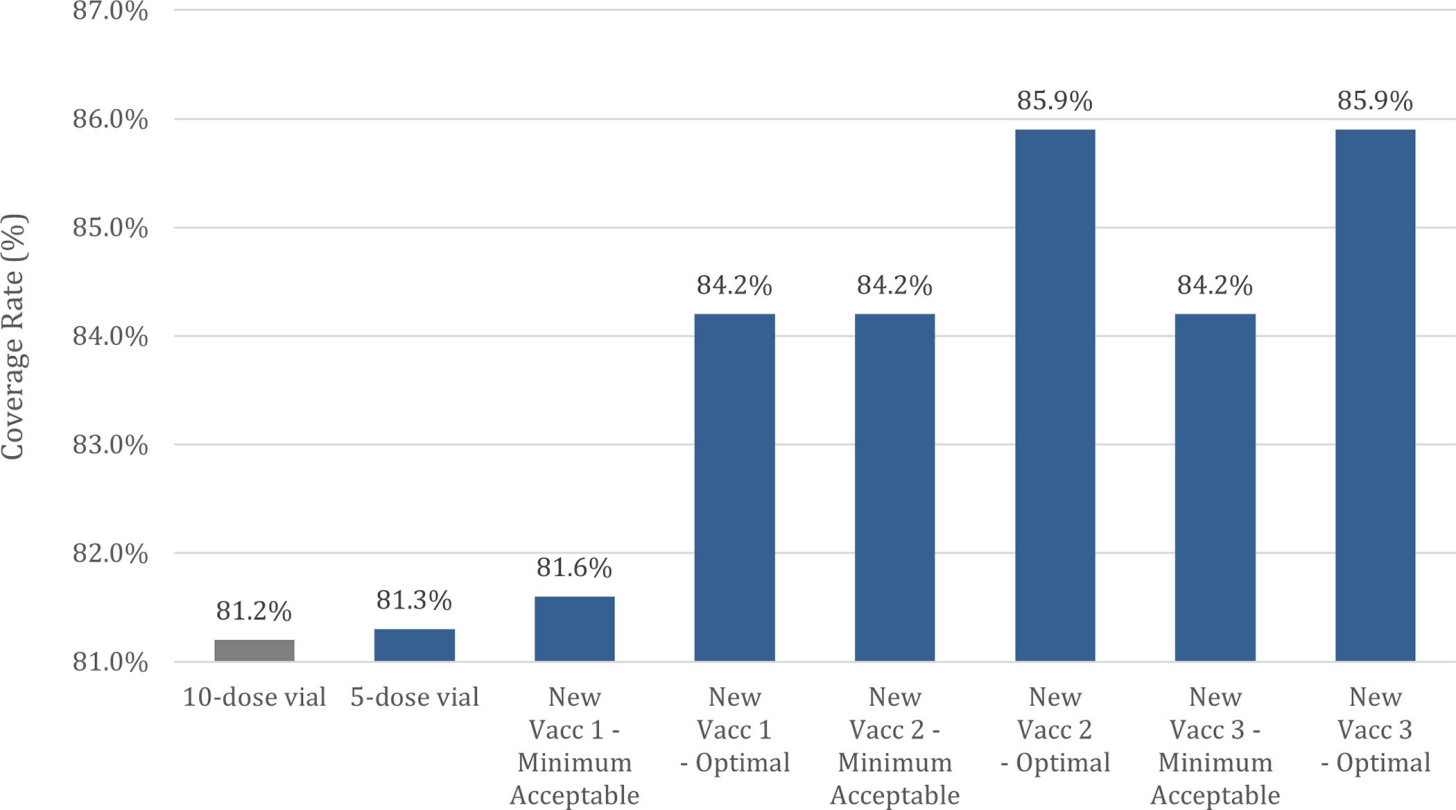
First-dose coverage rate for 10-dose vial and expected coverage rate for each new MR presentation in 82 low- and lower-middle income countries.

Our analysis indicated that each new vaccine technology would likely drive modest but meaningful improvements in vaccination coverage rate over the current 10-dose vial option. Shown in Fig 5, these improvements would translate to an increase of up to 3.0% when using a “minimum acceptable” presentation and up to 4.7% when using an “optimal” presentation. During the first year of deployment, these increases equate to up to 2.8 million additional people vaccinated when using a “minimum acceptable” presentation and up to 4.4 million additional people vaccinated when using an “optimal” presentation.

While these higher coverage rates could be achieved by full replacement of the current 10-dose vial, this would likely be an expensive option. As shown in Table 2, achieving 84.2% coverage by fully replacing the 10-dose vial with the “minimum acceptable” presentation of new Vaccine 2 would cost $228m per year, or $37 per additional individual vaccinated. An alternative would be to use a portfolio of vaccines, reaching as many individuals as possible with less expensive vaccines and targeting new presentations to individuals facing the barriers to vaccination that those presentations address. Table 2 summarizes the five lowest-cost portfolios that achieve this 84.2% coverage rate, with Portfolio 1 being the least costly option. By creating a portfolio with a large proportion of the baseline vaccine and small proportion of new Vaccine 2, the same coverage rate is achieved at a cost of $10 per additional person vaccinated instead of $37.

**Table 2:** Summary of the five lowest-cost portfolio options that achieve the global coverage rate of 84.2% possible under the assumption of “minimum acceptable” characteristics for the new vaccine technologies. All values indicate the average per year over a 10-year period that incorporates population growth. Annual Portfolio Cost, given as NPV, is the buyer-side cost and includes purchase price, service delivery cost, and amortized R&D or capital investments.

		Current Baseline Only	Portfolio 1	Portfolio 2	Portfolio 3	Portfolio 4	Portfolio 5	New Vaccine 2 Only
**Average Annual Demand** *Millions of doses*	*10-dose vial*	122.2	122.2	122.2	122.2	122.2	122.2	-
	*5-dose vial*	-	-	-	0.1	0.1	-	-
	*New Vaccine 1*	-	-	-	-	-	0.4	-
	*New Vaccine 2*	-	3.7	-	3.6	-	3.2	101.4
	*New Vaccine 3*	-	-	3.5	-	3.4	-	-
**Average Annual Portfolio Cost** *Million USD*		**$112**	**$144**	**$145**	**$152**	**$152**	**$161**	**$228**
**Average Annual Number of People Vaccinated** *Millions*		**83.1**	**86.2**	**86.2**	**86.2**	**86.2**	**86.2**	**86.2**
**Expected Coverage Rate**		**81.2%**	**84.2%**	**84.2%**	**84.2%**	**84.2%**	**84.2%**	**84.2%**

In general, we see the most cost-effective portfolios adhere to the following logic:

1. Maximize demand for the lowest-cost presentation, which in this case is the current 10-dose vial given our available data inputs
2. Supplement with *one* complementary vaccine technology, in this case the lowest cost of the new vaccine technologies that also drive increased coverage rates

Including at least one new technology raises the overall expected coverage rate (and therefore increases buyer benefit and buyer NPV), but adding a second new technology results in no gains in coverage and increased total cost. There are no gains in coverage because these new technologies target similar barriers to vaccination, and there is an increase in total cost due to the higher price per unit of the new vaccines, given lower volumes procured and the substantial fixed costs associated with each.

While in theory countries could create a more even split in global demand by building diverse individual country-level portfolios, economies of scale will likely drive them towards similar portfolio choices, especially since the three new technologies are relatively similar in the improved characteristics they target (e.g., thermostability and ease of administration). Splitting the same demand across multiple new technologies will lead to lower global demand volumes and likely higher per-dose prices for everyone.

**Fig 6:**
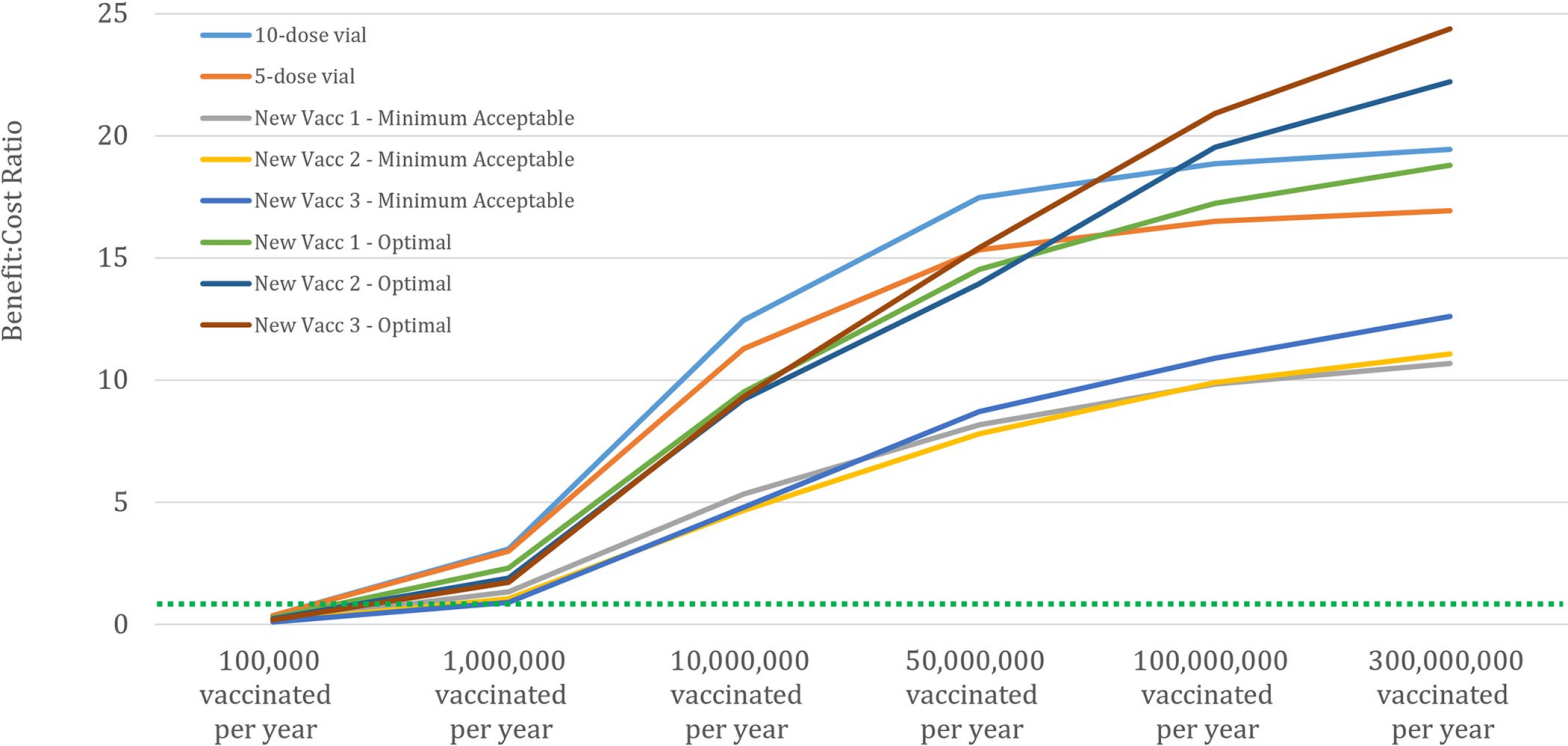
Estimated buyer benefit-cost ratio for each vaccine presentation at different levels of annual demand. The benefit-cost ratio calculations incorporate vaccine purchase price, service delivery costs, wastage, and economic value of vaccination. The green horizontal line corresponds to a ratio of 1, representing the minimum threshold for a “good” investment.

Related to this last point, we also investigated the minimum demand volume that would be needed for each presentation to become “profitable” to buyers (i.e., a ratio of buyer benefits to buyer costs that is greater than 1, which is equivalent to an NPV greater than $0), These estimates are shown Fig 6.

Buyer benefit-cost ratios (BCRs) are mixed at lower demand volumes. At the lowest increment of 100,000 people vaccinated per year, no product has a favorable BCR. This is because the expected purchase price at such low volumes exceeds the economic value that a vaccination provides. At 1 million people vaccinated per year, most presentations have flipped to a favorable BCR, but two of the new technologies remain unfavorable under the “minimum acceptable” set of assumed characteristics. At 10 million people per year vaccinated, all presentations have a *very* favorable BCR. However, given our target population size and the portfolio dynamics described in Table 2, this result further indicates that we are unlikely to see portfolios of more than two presentations, given the demand required for each vaccine to achieve a positive buyer BCR.

In summary, based on current data and projections, our analysis suggests that the MR vaccine market will likely benefit from the addition of one new vaccine technology in addition to the current 10-dose vial presentation. However, the market will likely not support additional entrants unless some fundamental aspect of the analysis changes; for example, if the new technologies begin to differentiate themselves in the barriers to vaccination they target, or if one technology achieves a cost breakthrough that makes it more affordable than the current 10-dose vial. Directly comparing specific MR technologies head-to-head will require more specific data, but this same analysis framework could be updated with more accurate data as those technologies progress through research and development.

## Discussion

### Contributions

The model described in this paper provides users with a simple, systematic and transparent way for evaluating vaccine-related investment scenarios. The model has several benefits:

1. *The model is grounded in a basic business approach for making investment decisions*. The cost-benefit analysis employed here will be familiar to nearly all investors from the private sector. It is a systematic approach for understanding the total costs and total benefits of any type of investment. By dollarizing all costs and benefits, users can easily compare all investment opportunities.
2. *The model incorporates the perspective of both the buyers and sellers*. No investment or purchase will happen unless both the buyer and seller believe they are benefiting from the transaction (i.e., the benefits outweigh the costs). By including the perspective of both the buyer and seller in the same model, it allows the user to answer the question, "Is my plan likely feasible?" For example, suppose a buyer would like to pay $2 per unit for 100 million units +/- 10 million units. The user could evaluate the feasibility of the scenario by using the model. Similarly, the model provides users with the opportunity to test "what-if scenarios." For example, suppose the above scenario was not feasible from the perspective of the seller of the vaccine. What would happen to the feasibility of the transaction if the buyer were able guarantee demand for 20 million units per year?
3. *The model incorporates the impact of uncertainty*. The model allows the user to add an uncertainty value to many of the inputs. Including uncertainty in the model was particularly important given that we expect most users will be interested in evaluating products still in development, when uncertainty is at its highest.
4. *The model integrates the concept of equity into the analysis*. As described above, the model allows users to value the benefit of vaccinating some populations (e.g., vulnerable populations, rural populations) differently than the benefit of vaccinating other populations (e.g., wealthy urban populations). This aspect of the model is particularly important when considering vaccine investments aimed at reaching harder to reach populations.
5. *The model is transparent*, *and the default inputs are modifiable*. The model was built in Excel largely to facilitate transparency and make assumptions explicit. The user has control over all values that are used in the cost-benefit analysis. While the model also includes ’default’ values for some variables (e.g., delivery costs) that are expected to be unknown by many of the users, these values can be easily overridden.
6. *The model can be used throughout the vaccine development cycle*. We expect that the accuracy of the inputs, and therefore the accuracy of the outputs, would improve as vaccine candidates move from one development phase to the next. One could imagine running the model during each development phase (e.g., *in vitro* screening, preclinical, Phase I, Phase II, Phase III, market) to determine which vaccine candidates to invest in or purchase.

Although there are several frameworks, approaches and models that provide one or more of the benefits described above, we are not aware of any other model that combines all these benefits into a single publicly available model. We believe that the outputs of the model would be useful to vaccine buyers and sellers, particularly those in the global health space.

### Assumptions and limitations

Our proposed methodology relies on several key assumptions to ensure conceptual simplicity and maintain tractability in an Excel-based model. These assumptions include:

1. *No funding constraints on vaccine demand*. It is beyond the scope of this model to forecast availability of funding for additional vaccine doses at the donor and country levels; estimates from the coverage model are taken as-is and treated as realized demand. We believe this assumption is well justified, since a major proposed use case for this model is to help vaccine buyers prepare, justify, and advocate for funding to cover changes in demand.
2. *As described above*, *vaccine prices are based on actual seller costs*. Our methodology assumes that the seller’s price is a function of their costs and perceived risks. We do not incorporate any competitive market tactics like loss leader pricing, cross-subsidization or other strategies through which a seller might choose to offer the vaccine below cost.
3. *Only individuals who are fully vaccinated contribute to DALYs averted and buyer benefit*. Individuals who are partially vaccinated, such as those who drop out after the first dose of a multi-dose regimen, are not incorporated into the DALY calculations and assumed to realize no health benefit.
4. *As described above*, *the model can perform only basic DALY calculations to estimate buyer benefit*. It is preferred for the user to input DALY figures from outside sources such as the Global Burden of Disease (GBD) Study database, which incorporates a more sophisticated set of disease- and geography-specific assumptions. The model’s built-in calculations are intended as a method of last resort for diseases or geographies that lack more detailed external data.
5. *Each vaccine in a portfolio can be accurately targeted to the individuals who benefit from its presence in the portfolio*. Using marginal increases in coverage rate to estimate vaccine demand inherently assumes that we can easily differentiate which people in the population are reachable with each of the vaccines, and target vaccine supply accordingly. In reality, targeting will be imperfect. A country will likely need to stock more of a new vaccine than initially estimated, as some of the new vaccine stock will incorrectly be channeled to individuals who could have been vaccinated with the status-quo vaccine presentation. As a result, the demand estimates produced by our model are likely a lower bound.

The above assumptions and limitations pertain to the methodology in general. Our example application to MR vaccine technologies involved several additional assumptions that are likely be important for users to consider for future applications. These assumptions include:

1. *Research and development costs for new MR technologies were not prorated across any other potential applications*. In other contexts, if a new technology has multiple immediate applications outside the analysis at hand, it may be wise for the user to prorate any initial research and development costs across these applications to increase the accuracy of the benefit cost ratio.
2. *The potential impact of new presentations on service delivery costs is fully realized*. For example, a heat stable MR vaccine has the *potential* to reduce cold chain costs. In reality, that benefit may not be realized if other vaccines continue to require cold storage. In our MR portfolio analysis, we assumed that these potential benefits were fully realized, but in other contexts the user should consider whether that assumption is realistic or helpful.
3. *The impact of new presentations on service delivery costs is applied equally across the entire target population*. In reality, we expect that a technology like MAP may be used to target remote populations that are more difficult–and more expensive–to reach. However, those costs would be incurred for any presentation used to reach that population. Therefore, in the model we assume an identical target population with the same *baseline* service delivery cost for each presentation (the service delivery cost impacts described in #2 above are still applied to this baseline service delivery cost).
4. *All presentations are available on the market at the same time*. MR technologies are in varying stages of development. To simplify the analysis, we assumed that all were available for production in Year 1.
5. *Only demand from LMIC countries is included in the analysis;* for many technologies, buyers and sellers, this assumption likely holds true. But some new technologies or presentations may experience significant demand from middle- or high-income countries. In these cases, a focus only on LMICs may impact the accuracy of unit price estimates, particularly in a portfolio where volumes per presentation can be low.
6. *Sellers will fill any funding gap for expected R&D costs of each vaccine technology*. Our analysis indicated a large gap between the expected R&D costs of MAP, dual chamber, and aerosol and currently committed funding. We assume that this gap will be filled largely by vaccine manufacturers rather than donors. This assumption results in lower up-front costs and higher unit prices for vaccine buyers, with minimal net impact on the final buyer NPV.

### Comparison to alternatives

To our knowledge, there are no publicly available models for systematically and transparently estimating the value and risk of a given vaccine-related investment scenario from the perspective of both buyers and sellers of vaccines. While there are publicly available tools and models that provide CBAs from the perspective of buyers, such as the OneHealth Tool [31], these tools are focused on existing products and may not be appropriate for products still in development. In addition, we are not aware of a publicly available model or tool that incorporates the concept of equity or monetizes the health benefit (e.g., DALYs averted) to facilitate easier comparisons between investment opportunities.

### Potential improvements

The model is grounded in a basic business approach for making investment decisions. For this reason, we do not believe the overall structure needs to be modified. However, we could imagine three major changes to the implementation that could improve the accuracy of the results and/or user experience:

1. *Build a more comprehensive library of default values*. The default values that exist within the model were collected through desk research and interviews with experts. However, the library we built was not comprehensive (i.e., there are not default values for each input) nor systematically cross-checked using multiple sources.
2. *Refine the user interface*. We consider the current version of the model to be a proof-of-concept that demonstrates the feasibility of conducting a CBA and portfolio analysis within an overarching structure. Additional work is needed to ensure that the user interface meets the needs of individuals using the model.
3. *Port the model from Excel to another software*. As mentioned above, Excel was chosen because it facilitated transparency in the calculations. Excel is also familiar to most potential users. Yet, the calculations that the model completes are complex relative to a typical spreadsheet model. There can be a lag in time between when the user starts the calculation process and when results are available. We can imagine some users would prefer a faster version of the model at the expense of some transparency.

## Conclusion

This paper describes how a model grounded in a cost-benefit approach can be used by both the buyers and sellers of vaccines to inform purchasing and investment decisions. By monetizing all inputs and outputs, the model allows the user to directly compare investment scenarios in a systematic and transparent manner that would be familiar to those working with, or within, a private sector organization. The model can be used to evaluate individual investments or purchases as well as optimize a portfolio of investments or purchases. By using the model throughout the vaccine development cycle, users can monitor the feasibility of their plan while refining their assumptions. Although investment and purchasing decisions are often based on more than simple cost-benefit analyses, we believe that the outputs of the model would be useful to vaccine buyers and sellers, particularly those in the global vaccine market. Finally, while we have chosen to focus our model on vaccines, the general approach could be applied to any health-related product or service.

## Supporting information

S1 File Excel model used for example application. (XLSM)

S2 File Overview of model inputs. (DOCX)
