# Supplementary material for: A model for estimating costs and benefits of new vaccine technologies from the perspective of both buyers and sellers
Source: PLoS One. 2023 Apr 5;18(4):e0283977. doi: 10.1371/journal.pone.0283977 (PMC10075404; doi:10.1371/journal.pone.0283977)
Supplement: S2 File — (DOCX) [file pone.0283977.s002.docx]

**Overview of key inputs, their relevancy for calculating costs and benefits for vaccine buyers and sellers, and data sources used for the example application to measles-rubella vaccine technologies**

|  | **Relevancy for calculating:** | | | | **Data sources used in measles-rubella example application** |
| --- | --- | --- | --- | --- | --- |
| **Key Input Parameters** | *Buyer Benefits* | *Buyer Costs* | *Seller Benefits* | *Seller Costs* |  |
| Vaccine efficacy rate | X |  |  |  | *For existing presentations*: U.S. Centers for Disease Control factsheet  *For new technologies:* interviews with the Bill & Melinda Gates Foundation (BMGF) CMC team |
| Duration of vaccine effectiveness (in years) | X |  |  |  | *For existing presentations*: U.S. Centers for Disease Control factsheet  *For new technologies:* interviews with BMGF CMC team |
| Annual disease incidence rate (%) in the target population | X |  |  |  | Global Burden of Disease (GBD) Results Tool |
| Average number of Disability-Adjusted Life Years (DALYs) per disease case | X |  |  |  | Global Burden of Disease (GBD) Results Tool |
| The economic value of a DALY among the target population | X |  |  |  | Robinson et al. Valuing Mortality Risk Reductions in Global Benefit-Cost Analysis. J Benefit Cost Anal. 2019. |
| Vaccination coverage rate (%) within the target population | X |  | X |  | Demographic and Health Survey (DHS) and other survey data at https://www.statcompiler.com/en/ |
| The size and annual growth rate of the target population | X | X | X | X | World Bank data from <https://data.worldbank.org/>  Indicators: Total Population and Population Growth |
| The number of doses needed for immunization |  | X | X | X | World Health Organization technical briefs and U.S. Centers for Disease control factsheets |
| Vaccine price per dose (USD) |  | X | X |  | Not Applicable; CBA calculations solve for price as a function of the other inputs, assuming seller NPV = 0 |
| The probability of technical and regulatory success (PTRS), per stage of vaccine development | X |  | X |  | Interviews with BMGF CMC team |
| Fixed initial investments, e.g., research and development and facility construction |  |  |  | X | Interviews with BMGF CMC team; Author analysis of available literature, chiefly:   - Plotkin et al. The complexity and cost of vaccine manufacturing – An overview. Vaccine. 2019. - Munira et al. A Cost Analysis of Producing Vaccines in Developing Countries. Vaccine. 2018 |
| Annual recurring expenditures, such as facility maintenance and ongoing regulatory approvals |  |  |  | X |  |
| Cost of Goods Sold (COGS, i.e., variable costs per vaccine unit produced) |  |  |  | X |  |
| Expected useful life for the manufacturing facility (in years) |  |  |  | X | Interviews with BMGF CMC team |
| Expected salvage value of facility assets (% of initial value) |  |  |  | X | General assumption of 10%; validated by BMGF CMC team |
| Average service delivery cost per dose |  | X |  |  | Author analysis of Immunization Costing Action Network (ICAN) cost catalogue available at [https://immunizationeconomics.org/](https://immunizationeconomics.org/ican-home/) |
| Expected closed- and open-vial wastage rates |  | X |  | X | Default WHO/Gavi assumptions from WHO Effective Vaccine Management Assistant tool. |
| Amount of prior and future grant funding invested in vaccine development, and/or production |  | X |  |  | Interviews with BMGF CMC team |
| Amount of prior and future loan funding, with average interest rate and repayment period. |  | X |  |  | Interviews with BMGF CMC team |
| Cost of Capital (%) used as discount rate for future benefits and costs | X | X | X | X | General assumption of 10% for both buyers and sellers; validated by BMGF CMC team |
| Overall time horizon (years) for CBA analysis | X | X | X | X | Interviews with BMGF CMC team;  10 years for MR example application |
